# Comparison of specimen extraction site and another site for protective loop ileostomy in laparoscopic low anterior rectal resection: a retrospective comparative study

**DOI:** 10.1007/s00423-023-02886-5

**Published:** 2023-04-13

**Authors:** Chao Liu, Jizhun Zhang, Leping Li, Li Zhang, Liang Shang, Yan Ma

**Affiliations:** 1 Department of Laser Cosmetic Clinic, Shandong Provincial Hospital Affiliated to Shandong First Medical University, Jinan, Shandong, 250021 China; 2 Department of Gastrointestinal Surgery, Shandong Provincial Hospital Affiliated to Shandong First Medical University, Jinan, Shandong, 250021 China

**Keywords:** Rectal cancer, Laparoscopic low anterior resection, Protective loop ileostomy, Ileostomy position, Specimen extraction site

## Abstract

**Background:**

Protective loop ileostomy is commonly performed in laparoscopic low anterior rectal resection to prevent the serious complications of anastomotic fistula. It is usually created at the right lower quadrant of the abdomen and another wound is required for stoma. The study aimed to evaluate the outcomes of ileostomy at the specimen extraction site (SES) and another site (AS) beside the auxiliary incision.

**Methods:**

A retrospective analysis was conducted on 101 eligible patients with pathologically diagnosed adenocarcinoma of the rectum from January 2020 to December 2021 in the study center. According to whether the ileostomy was at the specimen extraction site, patients were divided into SES group (40 patients) and AS group (61 patients). Clinicopathological characteristics, the intraoperative details, and postoperative outcomes of the two groups were measured.

**Results:**

Univariate analysis showed that the operative time was significantly shorter and the blood loss was significantly less in the SES group than in the AS group during laparoscopic low anterior rectal resection, the time to first flatus was significantly shorter, and the pain was significantly less in the SES group than in the AS group during ileostomy closure. The postoperative complications were similar in both groups. Multivariable analysis showed that ileostomy at the specimen extraction site was a significant factor influencing the operative time and blood loss of rectal resection, and influencing the pain and the time to first flatus during ileostomy closure.

**Conclusion:**

Compared to ileostomy at AS, protective loop ileostomy at SES was time-saving and less bleeding during laparoscopic low anterior rectal resection, and more quick to first flatus and less pain during stoma closure, and did not lead to more postoperative complications. The median incision of the lower abdomen and the left lower abdominal incision were both good sites for ileostomy.

## Introduction 

Globally, colorectal cancer ranks third in incidence and second in mortality. In China, there were about 560,000 new cases of colorectal cancer in 2020, of which 57.6% were rectal cancer patients, with a high proportion of low and middle patients [1]. Laparoscopic surgery in patients with rectal cancer resulted in similar safety, resection margins, completeness of resection, locoregional recurrence rate, disease-free survival rate, and overall survival rate to those of open surgery, and recovery was improved after laparoscopic surgery [2–5]. Laparoscopic low anterior resection has become the major treatment for low rectal cancer. However, anastomotic fistula is still an inevitable and important complication affecting the outcome of rectal cancer surgery. Studies found that anastomotic fistula after rectal cancer surgery not only increased the length of hospital stay and cost, but also related to local recurrence and low survival rate [6, 7].

Protective loop ileostomy was recommended as a protective diverting method for distal anastomosis in low anterior resection surgery, because of reducing anastomotic fistula rate and severe complications related to anastomotic fistula [8–10]. The traditional ileostomy was located in the right lower abdomen, close to the end of the ileum, but another wound was required for stoma beside the auxiliary incision [11]. Studies showed that stoma location had an impact on stoma complications and the incidence of surgical site infections [12, 13]. Some studies compared ileostomy with transverse colostomy, some proposed stoma via the specimen extraction site, and some investigated the feasibility of a loop ileostomy in the umbilical region or the left lower abdomen [14–18]. At present, there is no consensus on the location selection for the protective loop ileostomy. This retrospective study compared protective loop ileostomy at the specimen extraction site (SES) and at another site (AS) beside the auxiliary incision, to help surgeons make better decisions.

## Materials and methods

### Study design and population

A retrospective study of protective loop ileostomy was performed at Shandong Provincial Hospital Affiliated to Shandong First Medical University. The study included rectal cancer patients who underwent the first operation of laparoscopic low anterior resection following protective loop ileostomy and the second operation of ileostomy reversal from January 2020 to December 2021.

The included patients were pathologically diagnosed with adenocarcinoma of the rectum preoperatively, with a distance from the lower edge of the tumor to the anal verge ≤ 10 cm and the American Society of Anesthesiologists (ASA) score ≤ 3. Patients who had cancer metastasis, multiple cancers, conversion to open surgery, and a history of previous colorectal surgery and those who underwent emergency surgery were excluded. Patients with incomplete medical records of resection and closure were also excluded.

All patients underwent a preoperative assessment, including physical and laboratory examinations, cardiopulmonary function tests, colonoscopy with biopsy, total abdominal enhanced computed tomography scan, and pelvic magnetic resonance imaging. According to the preoperative evaluation of the American Joint Committee on Cancer (AJCC) staging system (8th edition), some patients underwent preoperative neoadjuvant chemoradiotherapy. All patients received the same perioperative therapy regimen. Bowel preparation was needed, and the nasogastric tube was not used. All patients underwent laparoscopic low anterior resection and Dixon anastomosis with total mesorectal excision (TME). All patients had R0-resection operation in this study, and pathologies of all excised specimens indicated R0-resection. A 20–24-Fr drainage tube was routinely placed in the pelvic area during surgery, and removed 3–7 days after surgery according to the amount of drainage fluid.

### Data collection and grouping

All the data were obtained from the patients’ medical records, and the study was approved by the ethics committee of the study center. The collected data included four parts: first, demographics including age, gender, body height, body weight, body mass index (BMI), smoking habits, hypertension, diabetes mellitus, preoperative neoadjuvant chemoradiotherapy, ASA score, preoperative serum hemoglobin, preoperative serum albumin, and preoperative serum prealbumin; second, clinicopathological characteristics including distance of tumor to the anal verge and AJCC TNM stage; third, surgical related factors including operative time, intraoperative blood loss, operative complications, nutritional risk score, and postoperative pain score; and fourth, details of the stoma closure such as interval times before closure, peristomal adhesion extent, and time to first flatus.

The nutritional risk score was calculated using the nutritional risk screening (NRS 2002) method, and the postoperative pain score was calculated using the Numerical Rating Scale [19, 20]. The peristomal adhesion extent was classified into four degrees [21]: nil, mild (no obvious adhesion or light adhesions, which can easily be separated by blunt dissection), moderate (adhesions where blunt dissection is possible but sharp dissection necessary, with vascularization), and severe (lysis of the adhesions is possible by sharp dissection only, organs are strongly attached, and organ damage is hard to prevent). Operative complications included surgical site infection (SSI), anastomotic fistula, ileus, urinary tract infection, pneumonia, thrombotic events, and stoma-related complications such as stoma stricture, stoma retraction, stoma bleeding, stoma prolapse, stoma necrosis, skin irritation, and parastomal hernia.

According to whether the protective loop ileostomy was at the specimen extraction site (SES) or at another site (AS) beside the auxiliary incision, patients were divided into two groups, the SES group and the AS group. The demographics, clinicopathological characteristics, surgical related factors, and details of the stoma closure procedure were compared and analyzed.

### Statistical analysis

All analyses were performed using IBM SPSS Statistics for Windows version 25 (IBM Corp., Armonk, NY, USA). Mann-Whitney *U* tests were used for non-normally distributed continuous variables, which were shown as the median and range. Categorical variables were analyzed using the *χ*^2^ test or Fisher’s exact test. Multiple logistic regression was used to perform multivariate analysis of the statistically significant variables in the univariate analysis, and the results are expressed as odds ratios (ORs) and 95% confidence intervals (95% CIs). *P* values of < 0.05 were considered statistically significant.

## Results

### *Patients’ demographics and clinicopathological characteristics*

**Fig. 1:**
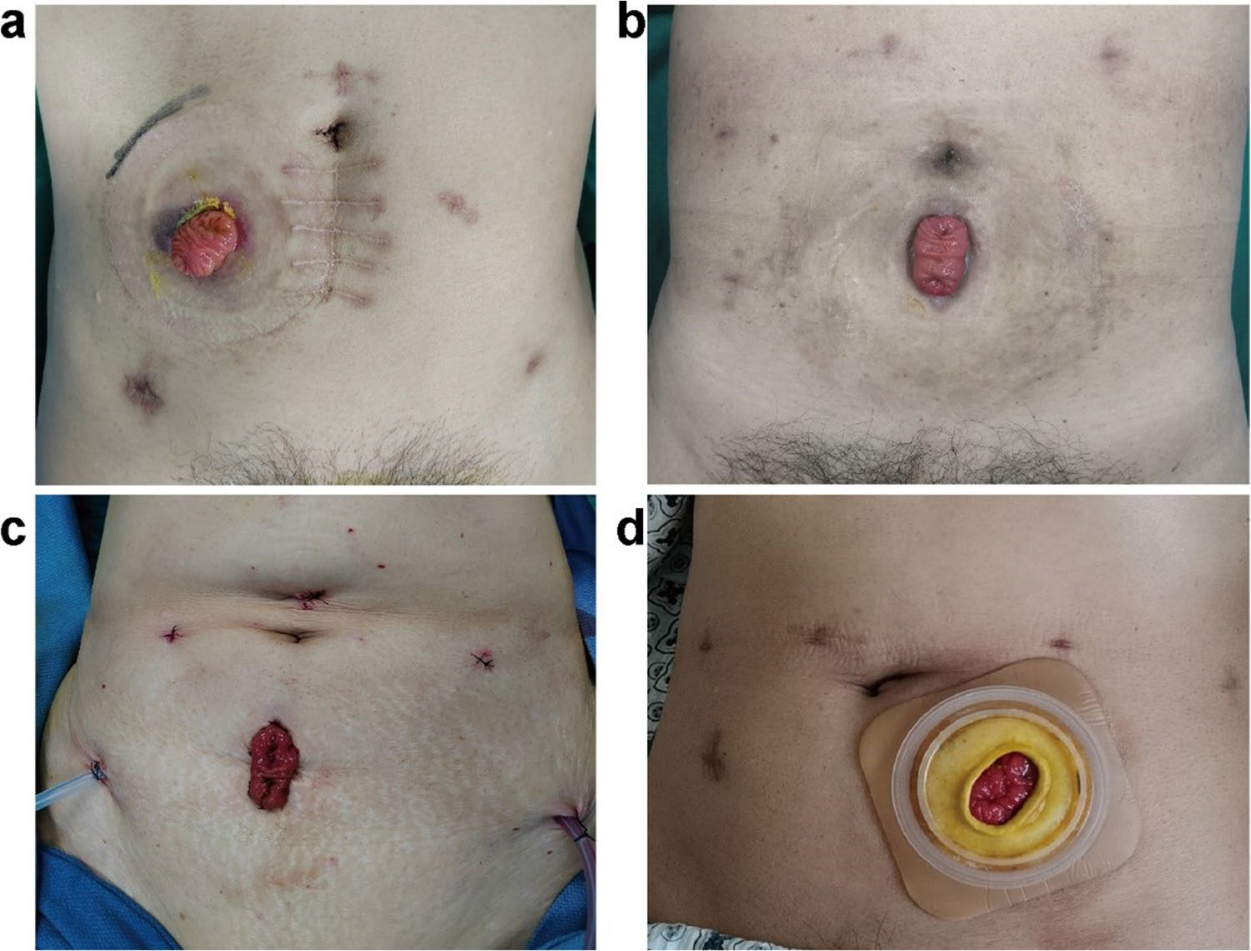
Different sites of protective loop ileostomy. **a** Protective loop ileostomy at the right lower abdomen beside median incisions of the lower abdomen (AS group). **b**, **c** Protective loop ileostomy at the specimen extraction site (SES) located in the median incision of the lower abdomen. **d** Protective loop ileostomy at the specimen extraction site (SES) located in the left lower abdominal incision

A total of 101 patients who underwent laparoscopic low anterior rectal resection following protective loop ileostomy at Shandong Provincial Hospital Affiliated to Shandong First Medical University from January 2020 to December 2021 were included in this study. The SES group consisted of 40 patients (19 males, 21 females), while the AS group consisted of 61 patients (36 males, 25 females). The sites of ileostomy in the AS group were all at the right lower abdomen beside median incisions of the lower abdomen. The sites of ileostomy in the SES group included stoma at median incision of the lower abdomen (19 patients), stoma at left lower abdominal incision (15 patients), and stoma at right lower abdominal incision (6 patients). Ileostomy locations are shown in fig. 1. The baseline demographics and the clinicopathological characteristics are summarized in Table 1. No significant differences (*P* > 0.05) were detected between the SES and AS groups with respect to age, gender, body height, body weight, BMI, smoking habits, hypertension, diabetes mellitus, neoadjuvant therapy, ASA score, distance of tumor to anal verge, and AJCC TNM stage.

**Table 1:** The baseline demographics and the clinicopathological characteristics

	SES (*n* = 40)^a^	AS (*n* = 61)^a^	*P* value
Gender, male/female	19 (47.5%)/21 (52.5%)	36 (59.0%)/25 (41.0%)	0.256
Ages, years	62 (51–67)	62 (55–67)	0.564
Body height, cm	166 (157–171)	165 (158–169)	0.682
Body weight, kg	67.3 (57.5–81.6)	67.0 (60.0–76.5)	0.964
BMI, kg/m^2^	24.2 (22.7–27.4)	24.3 (22.5–27.0)	0.961
Smoking habits	8 (20.0%)	20 (32.8%)	0.160
Hypertension	14 (35.0%)	24 (39.3%)	0.659
Diabetes mellitus	5 (12.5%)	11 (18.0%)	0.456
Neoadjuvant therapy	8 (20.0%)	7 (11.5%)	0.239
ASA score 2 3	34 (85.0%) 6 (15.0%)	45 (73.8%) 16 (26.2%)	0.181
Distance of tumor to anal verge, cm	6 (5–9)	5 (5–8)	0.138
Pathological T stage			0.270
pT1	2 (5.0%)	1 (1.6%)	
pT2	13 (32.5%)	16 (26.2%)	
pT3	17 (42.5%)	37 (60.7%)	
pT4a	8 (20.0%)	7 (11.5%)	
pT4b	0	0	
Pathological N stage			0.854
pN0	24 (60.0%)	38 (62.3%)	
pN1a	3 (12.5%)	5 (8.2%)	
pN1b	5 (7.5%)	11 (18.0%)	
pN1c	2 (3.0%)	1 (1.6%)	
pN2a	3 (5.9%)	3 (4.9%)	
pN2b	3 (5.9%)	3 (4.9%)	
Pathological M stage			
pM0	40 (100%)	61 (100%)	
Pathological TNM stage (AJCC 8th edition)			0.310
I	12 (30.0%)	13 (21.3%)	
IIA	4 (10.0%)	18 (29.5%)	
IIB	3 (7.5%)	3 (4.9%)	
IIC	0	0	
IIIA	3 (7.5%)	3 (4.9%)	
IIIB	13 (32.5%)	19 (31.1%)	
IIIC	5 (12.5%)	5 (8.2%)	

Abbreviation: *SES*, specimen extraction site; *AS*, another site; *BMI*, body mass index; *ASA*, American Society of Anesthesiologists; *TNM*, tumor-node-metastasis; *AJCC*, American Joint Committee on Cancer

^a^Values are shown as *n* (%) or median (interquartile range)

### Perioperative data of laparoscopic low anterior rectal resection following protective loop ileostomy

All surgeries were performed successfully by experienced surgeons using a standard procedure. The perioperative data of laparoscopic low anterior rectal resection following protective loop ileostomy are shown in Table 2. The median operative time in the SES group was significantly shorter than that in the AS group (170 vs. 190 min, *P* = 0.030). The proportion of patients whose intraoperative blood loss > 50 ml in the SES group was significantly less than that in the AS group (5.0% vs. 44.3%, *P* < 0.001). No significant difference was detected between the two groups in operative complications (17.5% vs. 26.2%, *p* = 0.306), postoperative fasting time (*p* = 0.441), overall length of hospital stay (9 vs. 9 days, *p* = 0.763), NRS 2002 score (*p* = 0.118), pain-rating scale (*p* = 0.255), preoperative hemoglobin (*p* = 0.950), preoperative serum albumin (*p* = 0.182), and preoperative serum prealbumin (*p* = 0.433).

**Table 2:** Perioperative data of laparoscopic low anterior resection of rectal cancer following protective loop ileostomy

	SES (*n* = 40)^a^	AS (*n* = 61)^a^	*P* value
Operative time, min	170 (160–209)	190 (170–236)	0.030*
Intraoperative blood loss			< 0.001*
≤ 50 ml > 50 ml	38 (95.0%) 2 (5.0%)	34 (55.7%) 27 (44.3%)	
Postoperative fasting time, days	4 (3–5)	4 (3–5)	0.441
Operative complications	7 (17.5%)	16 (26.2%)	0.306
Overall length of hospital stay, days	9 (8–11)	9 (8–12)	0.763
NRS 2002 score	2 (2–3)	3 (2–3)	0.118
Pain-rating scale			0.255
2 3 4	36 (90.0%) 4 (10.0%) 0	48 (78.7%) 11 (18.0%) 2 (3.3%)	
Preoperative hemoglobin, g/l	128 (119–144)	131 (117–143)	0.950
Preoperative serum albumin, g/l	39.4 (38.1–42.4)	39.3 (37.2–41.7)	0.182
Preoperative serum prealbumin, mg/l	217 (196–255)	211 (178–249)	0.433

Abbreviation: *SES*, specimen extraction site; *AS*, another site; *NRS*, nutritional risk screening

^**a**^Values are shown as *n* (%) or median (interquartile range)

^*^P value is statistically significant

In multivariate analysis (Table 3), the significant independent factors influencing the operative time (cutoff value 180 min) of laparoscopic low anterior resection of rectal cancer following protective loop ileostomy included ileostomy at SES (OR 0.255, 95% CI = 0.071–0.919, *P* = 0.037) and BMI (OR 1.181, 95% CI = 1.004–1.388, *P* = 0.044). And as shown in Table 4, ileostomy at SES (OR 0.073, 95% CI = 0.016–0.343, *P* = 0.001) was also a significant independent factor influencing the proportion of patients whose intraoperative blood loss > 50 ml in operation.

**Table 3:** Multivariate analysis for operative time of laparoscopic low anterior resection of rectal cancer following protective loop ileostomy

	OR	95% CI	*P* value
Gender			
Male Female	3.720 1	0.989–13.988 Reference	0.052
BMI	1.181	1.004–1.388	0.044*
Distance of tumor to anal verge	0.784	0.570–1.078	0.134
The site of ileostomy			
SES AS	0.255 1	0.071–0.919 Reference	0.037*
Pathological TNM stage (AJCC 8th edition)			
I	0.795	0.086–7.376	0.840
IIA	0.280	0.020–3.928	0.345
IIB	0.219	0.008–5.764	0.363
IIIA	0.540	0.030–9.753	0.676
IIIB	0.695	0.087–5.576	0.732
IIIC	1	Reference	

Abbreviation: *OR*, odds ratio; *CI*, confidence interval; *BMI*, body mass index; *SES*, specimen extraction site; *AS*, another site; *TNM*, tumor-node-metastasis; *AJCC*, American Joint Committee on Cancer

^*^P value is statistically significant

**Table 4:** Multivariate analysis for intraoperative blood loss of laparoscopic low anterior resection of rectal cancer following protective loop ileostomy

	OR	95% CI	*P* value
Age	1.032	0.983–1.083	0.202
Gender			
Male Female	1.101 1	0.376–3.229 Reference	0.860
BMI	1.039	0.910–1.186	0.571
The site of ileostomy			
SES AS	0.073 1	0.016–0.343 Reference	0.001*
Distance of tumor to anal verge	0.985	0.775–1.251	0.901
Preoperative serum albumin	0.852	0.715–1.016	0.075

Abbreviation: *OR*, odds ratio; *CI*, confidence interval; *BMI*, body mass index; *SES*, specimen extraction site; *AS*, another site

^*^P value is statistically significant

### Details of stoma closure

The stoma closure procedure was performed under general anesthesia with a peristomal skin incision, release the adhesion around the stoma, stoma resection, and intestinal anastomosis. The fascia and skin were closed. All surgeons applied the same procedure for closure. The details of stoma closure are shown in Table 5. The time to first flatus in the SES group (median 3 days, interquartile range 2–4 days) was significantly shorter than that in the AS group (median 3 days, interquartile range 3–5 days) (*P* = 0.008). The pain-rating scale score in the SES group (score 2, 97.5%; score 3, 2.5%) was significantly lower than that in the AS group (score 2, 83.6%; score 3, 16.4%) (*P* = 0.046). No significant difference was detected between the two groups in the interval time between two operations (*p* = 0.164), operative time (95 vs. 95 min, *p* = 0.884), intraoperative blood loss (*p* = 0.104), operative complications (7.5% vs. 8.2%, *p* = 0.899), postoperative fasting time (*p* = 0.313), overall length of hospital stay (8 vs. 9 days, *p* = 0.540), NRS 2002 score (*p* = 0.187), peristomal adhesion extent (*p* = 0.062), preoperative hemoglobin (*p* = 0.224), preoperative serum albumin (*p* = 0.797), and preoperative serum prealbumin (*p* = 0.091).

**Table 5:** Details of stoma closure

	SES (*n* = 40)^a^	AS (*n* = 61)^a^	*P* value
Interval time, months	5 (3–6)	6 (4–7)	0.164
Postoperative chemotherapy or chemoradiotherapy	28 (70.0%)	50 (82.0%)	0.161
Operative time, min	95 (75–125)	95 (74–120)	0.884
Intraoperative blood loss			0.104
≤ 20 ml > 20 ml	23 (57.5%) 17 (42.5%)	25 (41.0%) 36 (59.0%)	
Peristomal adhesion extent			0.062
Nil Mild Moderate Severe	0 33 (82.5%) 4 (10.0%) 3 (7.5%)	0 37 (60.7%) 16 (26.2%) 8 (13.1%)	
Postoperative fasting time, days	4 (3–4)	4 (3–5)	0.313
Time to first flatus, days	3 (2–4)	3 (3–5)	0.008*
Operative complications	3 (7.5%)	5 (8.2%)	0.899
Overall length of hospital stay, days	8 (7–10)	9 (7–10)	0.540
NRS 2002 score	2 (2–3)	2 (2–3)	0.187
Pain-rating scale			0.046*
2 3	39 (97.5%) 1 (2.5%)	51 (83.6%) 10 (16.4%)	
Preoperative hemoglobin, g/l	131 (119–140)	128 (116–136)	0.224
Preoperative serum albumin, g/l	40.1 (37.9–42.1)	39.9 (38.0–41.3)	0.797
Preoperative serum prealbumin, mg/l	256 (218–304)	238 (206–274)	0.091

Abbreviation: *SES*, specimen extraction site; *AS*, another site; *NRS*, nutritional risk screening

^a^Values are shown as *n* (%) or median (interquartile range)

^*^P value is statistically significant

In multivariate analysis (Table 6), the significant independent factors influencing the first flatus time (cutoff value 3 days) after the surgery of ileostomy closure included ileostomy at SES (OR 0.212, 95% CI = 0.066–0.686, *P* = 0.010) and preoperative hemoglobin (OR 1.041, 95% CI = 1.001–1.083, *P* = 0.044). And as shown in Table 7, ileostomy at SES (OR 0.064, 95% CI = 0.005–0.766, *P* = 0.030) was also a significant independent factor influencing the pain-rating scale score after operation.

**Table 6:** Multivariate analysis for the time to first flatus after the surgery of ileostomy closure

	OR	95% CI	*P* value
The site of ileostomy			
SES AS	0.212 1	0.066–0.686 Reference	0.010*
Preoperative hemoglobin	1.041	1.001–1.083	0.044*
NRS 2002 score	1.439	0.802–2.584	0.223
Operative complications			
No Yes	0.229 1	0.020–2.583 Reference	0.233
Intraoperative blood loss	0.996	0.975–1.018	0.728

Abbreviation: *OR*, odds ratio; *CI*, confidence interval; *SES*, specimen extraction site; *AS*, another site; *NRS*, nutritional risk screening

^*^P value is statistically significant

**Table 7:** Multivariate analysis for pain-rating scale score after the surgery of ileostomy closure

	OR	95% CI	*P* value
The site of ileostomy			
SES AS	0.064 1	0.005–0.766 Reference	0.030*
NRS 2002 score	1.762	0.953–3.260	0.071
Operative complications			
No Yes	0.170 1	0.019–1.552 Reference	0.116
Operative time	0.972	0.941–1.004	0.084
Time to first flatus	0.911	0.601–1.382	0.661
Preoperative serum albumin	1.207	0.965–1.509	0.099

Abbreviation: *OR*, odds ratio; *CI*, confidence interval; *SES*, specimen extraction site; *AS*, another site; *NRS*, nutritional risk screening

^*^P value is statistically significant

## Discussion

At present, laparoscopic anterior resection of rectal cancer is the main surgical method for the treatment of middle and low rectal cancer [4, 22], but anastomotic fistula is still an unavoidable serious postoperative complication [7]. In order to reduce the incidence of anastomotic fistula and eliminate serious consequences caused by anastomotic fistula, protective loop ileostomy is commonly performed in laparoscopic low anterior rectal resection [10]. In a retrospective study, 89% patients who underwent rectal cancer resections with the formation of an anastomosis received a protective stoma in Australia and New Zealand [23].

Although protective loop ileostomy is widely used in laparoscopic anterior resection of rectal cancer, we need to pay attention to its disadvantages. First, a second operation is needed to close the ileostomy, but the ileostomy is at the risk of developing into a permanent stoma in cases of anastomotic leakage, local recurrence, and metastasis [24–26]. Second, the quality of life is reduced because patients would be disturbed by the smell or leakage of feces [27]. Third, the complications related to ileostomy such as stoma retraction, stoma prolapse, stoma necrosis, stoma stricture, stoma bleeding, skin irritation, and parastomal hernia could bring huge suffering to patients [28]. So it is important to master indications and methods of protective loop ileostomy better.

Protective loop ileostomy is usually created at the right lower quadrant of the abdomen because of its vicinity to the ileocecal valve. In the laparoscopic procedure, however, another wound is required for stoma beside the auxiliary incision, resulting in a scar after closure [29]. To achieve a more minimally invasive effect, more and more studies attempted to make a stoma at the specimen extraction site (also as the auxiliary incision) which is located in the left or right lower quadrant of the abdomen, even the periumbilical area [15, 16, 18, 30]. In our center, we also tried to make protective loop ileostomy at the specimen extraction site or auxiliary incision after laparoscopic low anterior rectal resection. Preoperative marking of the stoma site and education by a trained enterostomal therapist was needed [13, 31]. The usual auxiliary incisions were the median incision of the lower abdomen and the left lower abdominal incision, which were also the sites of ileostomy. Although the median incision of the lower abdomen is often used as an auxiliary incision, it is not usually used as the ileostomy sit, but we found it a good site of ileostomy in this study. First, the midline incision of the lower abdomen is useful for the specimen extraction of rectal cancer and the clipping of the sigmoid mesangium. Second, during closure of the ileostomy, the tension of median incision in the lower abdomen is not tight and is conducive to closing the abdominal wall defect at the stoma. Third, midline incision is also more convenient to extend for patients with severe pelvic adhesions than other incisions. Fourth, compared with the right lower ventral stoma, the median ventral stoma was equally convenient for ostomy bag replacement and did not increase leakage rate.

To evaluate the outcomes of ileostomy at the specimen extraction site (SES) and another site (AS) beside the auxiliary incision, a retrospective analysis was conducted on 101 eligible patients in this study. The median operative time of rectal resection surgery in the SES group was significantly shorter than that in the AS group (170 vs. 190 min, *P* = 0.030). The proportion of patients with intraoperative blood loss > 50 ml in the SES group was significantly less than that in the AS group (5.0% vs. 44.3%, *P* < 0.001). And in multivariate analysis, ileostomy at SES was not only a significant independent factor (OR 0.255, 95% CI = 0.071–0.919, *P* = 0.037) influencing the operative time but also a significant independent factor (OR 0.073, 95% CI = 0.016–0.343, *P* = 0.001) influencing the proportion of patients whose intraoperative blood loss > 50 ml in operation. It suggested that ileostomy at SES was more time-saving and less invasive than at AS. This phenomenon might be attributed to that one incision was reduced. Less incision often means less trauma, less bleeding, and less time of operation.

BMI was another significant independent factor (OR 1.181, 95% CI = 1.004–1.388, *P* = 0.044) influencing the operative time of rectal cancer. It is often more difficult to operate on obese patients than thin patients either in excision or in ileostomy. The length of incision at SES was 5–8 cm, it is a bit longer than the incision needed for a normal stoma, and the operator needed to close a part of the incision to create a stoma, but it is convenient for obese patients because of sufficient exposure in suturing. Although there is a risk of stool pollution on incision at SES, no significant difference was detected between the two groups in operative complications (*p* = 0.306). Besides, the method of ileostomy was also an important factor affecting the operation time; in our previous research at the center, we found that the one-stitch method of protective loop ileostomy exhibited more advantages than the traditional method, notably in BMI obesity patients [11, 32].

The univariate analysis of stoma closure data showed that the time to first flatus in the SES group was significantly shorter than that in the AS group (*P* = 0.008), and the pain-rating scale score in the SES group was significantly lower than that in the AS group (*P* = 0.046). In multivariate analysis, ileostomy at SES was not only a significant independent factor (OR 0.212, 95% CI = 0.066–0.686, *P* = 0.010) influencing the first flatus time but also a significant independent factor (OR 0.064, 95% CI = 0.005–0.766, *P* = 0.030) influencing the pain-rating scale score after the surgery of ileostomy closure. It suggested that the recovery of gastrointestinal function was more quick and the pain was less in stoma closure at SES than at AS. The less pain might attribute to the less tension of incision, because of the reduction of a scar on the abdominal wall and increased abdominal wall ductility. And less pain helped patients move early, thus contributing to recovery of gastrointestinal function. Although reduction of an incision might reduce abdominal adhesions, no significant difference on peristomal adhesion extent was detected between two groups (*p* = 0.062).

Nevertheless, the present study has some limitations. It is a retrospective analysis and a single-center study; therefore, it has some inherent biases. The ileostomy method selection may be objective and was based on the operative surgeons. And the data on the quality of life or patient satisfaction with the stoma sites was deficient.

## Conclusion

In conclusion, the present study showed that compared to ileostomy at AS, protective loop ileostomy at SES was time-saving and less bleeding during laparoscopic low anterior rectal resection, and more quick to first flatus and less pain during stoma closure, and did not lead to more postoperative complications. The median incision of the lower abdomen and the left lower abdominal incision were both good sites for ileostomy.

## Data Availability

The data used and analyzed during the current study are available from the corresponding author on reasonable request.
